# Mitochondrial Bioenergetics and Dynamism in the Failing Heart

**DOI:** 10.3390/life11050436

**Published:** 2021-05-12

**Authors:** Giampaolo Morciano, Veronica Angela Maria Vitto, Esmaa Bouhamida, Carlotta Giorgi, Paolo Pinton

**Affiliations:** 1 Maria Cecilia Hospital, GVM Care&Research, 48033 Cotignola, Italy; 2 Laboratory for Technologies of Advanced Therapies (LTTA), Section of Experimental Medicine, Department of Medical Sciences, University of Ferrara, 44121 Ferrara, Italy; vttvnc@unife.it (V.A.M.V.); bhmsme@unife.it (E.B.); carlotta.giorgi@unife.it (C.G.)

**Keywords:** mitochondria, heart failure, bioenergetics, mitochondrial dynamics

## Abstract

The heart is responsible for pumping blood, nutrients, and oxygen from its cavities to the whole body through rhythmic and vigorous contractions. Heart function relies on a delicate balance between continuous energy consumption and generation that changes from birth to adulthood and depends on a very efficient oxidative metabolism and the ability to adapt to different conditions. In recent years, mitochondrial dysfunctions were recognized as the hallmark of the onset and development of manifold heart diseases (HDs), including heart failure (HF). HF is a severe condition for which there is currently no cure. In this condition, the failing heart is characterized by a disequilibrium in mitochondrial bioenergetics, which compromises the basal functions and includes the loss of oxygen and substrate availability, an altered metabolism, and inefficient energy production and utilization. This review concisely summarizes the bioenergetics and some other mitochondrial features in the heart with a focus on the features that become impaired in the failing heart.

## 1. An overview of Mitochondrial Bioenergetics and Function in the Healthy Heart

Over recent years, mitochondrial dysfunction was recognized as the hallmark of manifold heart diseases (HDs) in their onset and development [1,2]. Despite the advancement of specific prevention guidelines and relevant therapeutic strategies, HD remains the main cause of death in Western countries [3]. Mitochondria occupy 30% of the total volume of cardiomyocytes and are localized in three different areas among cardiac fibers [4]; these structures are highly dynamic during all phases of heart development and exhibit continuous changes in terms of their bioenergetics and biology during cardiomyocyte differentiation [5]. This mitochondrial abundance is justified by the high-energy supply required by the heart that is provided by mitochondria, defined as “the powerhouse of the cells”. In fact, via oxidative phosphorylation (OXPHOS), mitochondria generate 95% of the adenosine triphosphate (ATP) needed to maintain cardiac activities [6]. In cardiac tissue, the principal source of energy is generated by fatty acid oxidation (FAO), which occurs in the mitochondrial matrix. The products obtained by beta-oxidation enter the Krebs cycle and are ultimately utilized by the electron transport chain (ETC), which takes place along the inner mitochondrial membrane (IMM), and this phenomenon creates a proton electrochemical gradient and consequently generates the mitochondrial transmembrane potential (ΔΨ_m_). Under physiological conditions, the ΔΨ_m_ is −180 mV across the IMM and thus acts as a driving force for cations, such as calcium (Ca^2+^), which is considered the most important second messenger in cells. Mitochondrial Ca^2+^ plays a crucial role in controlling cell physiopathology and cell fate [7]. In the heart, its importance is highlighted by the fact that it is fundamental for the contractile function of cardiomyocytes [8]; indeed, excitation and contraction are paired to cardiomyocyte depolarization and Ca^2+^ fluxes. Concomitant with an action potential, the sarcolemma depolarizes, and Ca^2+^ enters a dedicated area across the sarcolemma through L-type voltage-operated calcium channels (VOCCs). Here, the Ca^2+^ concentration increases from very low levels (nanomolar ranges) to approximately 10 µM to give rise to the so-called Ca^2+^ sparklet. These Ca^2+^ sparklets induce ryanodine receptor 2 (RyR2) opening and Ca^2+^ release from the main store inside cells, the sarcoplasmic reticulum (SR), which might generate higher Ca^2+^ sparks into the cytosol and thus allow cell shortening and blood pumping in the heart.

Mitochondrial Ca^2+^ also leads to the generation of ATP via the activation of dehydrogenases [9] and increasing the activity of the ATP synthase [10] when muscles are stimulated. ATP cycling, as well as Ca^2+^, constitutes a key point for the control of both muscle contraction and relaxation. Indeed, ATP is used by the sarcoendoplasmic reticulum Ca^2+^-ATPase cardiac isoform 2a (SERCA2a) to remove Ca^2+^ from the cytosol in the diastolic phase to ensure muscle relaxation and the correct Ca^2+^ reuptake into the SR for the next muscle contraction. Thousands of these events occur with each action potential. Although other systems of Ca^2+^ removal exist in the cells, such as Plasma membrane Ca^2+^ ATPase (PMCA) and Na^+^/Ca^2+^ exchanger (NCX), SERCA2a handles about 75% of cytosolic Ca^2+^.

Ca^2+^ is also responsible for activating cell death pathways. Indeed, it is widely reported that persistent accumulation of Ca^2+^ into the cytosol and mitochondrial Ca^2+^ overload trigger the opening of the mitochondrial permeability transition pore complex (PTPC), a biological event that, if sustained, leads to cell death [11,12].

An important consequence of mitochondrial function is reactive oxygen species (ROS) production [13]. Indeed, ROS are physiologically generated by the ETC via the reduction of oxygen to superoxide and by the Krebs cycle in the mitochondrial matrix. Although a balance exists between ROS production and scavenging systems and that ROS regulate some physiological processes, a significant dysregulation may occur in or as a consequence of diseases; indeed, ROS bursts can damage proteins, lipids, and mitochondrial DNA (mtDNA), which trigger inflammation and ultimately cell death as well as PTPC opening [14].

Heart failure (HF) is a severe condition for which there is currently no cure, and only medications are administered to maintain a patient’s life as normally as possible. Approximately 30 million people worldwide are suffering from HF, and these patients need new therapies that address the disease rather than only providing symptom relief. The clinical scenario is represented by two main conditions: either a reduction of left ventricular (LV) ejection fraction, named as heart failure with reduced ejection fraction (HFrEF) [15], which results in an enlarged LV that cannot contract as it should, or a preserved LV ejection fraction (HFpEF), where the LV preserves its ability to contract but, being less than the necessary volume of blood which enters in the left chamber during an improper diastole, it does not meet the body’s requirement. Mitochondria, intracellular Ca^2+^ and ATP cycling not by chance are associated with significant alterations during HF, and understanding these changes might have a potential impact on the search for new pharmacological treatments. Two of the main intracellular alterations in HF are the elevated cytosolic Ca^2+^ levels in diastole and its decrease in availability in the SR during systole. This leads to an inefficient cycle of excitation contraction coupling (ECC). Several studies in human and animal models have associated these defects in Ca^2+^ cycling to SERCA2a dysfunction [16,17] in terms of reduced expression and impaired regulation [18].

HF develops through stages of cardiac adaptation in terms of macroscopic (e.g., hypertrophy) and microscopic (e.g., metabolism) changes. During cardiac hypertrophy, a metabolic shift from FAO to glycolysis occurs [19]. This finding is supported by a seminal paper that, using the carbon isotope labelling techniques [20], showed the importance of the glycolytic pathway in producing ATP in hypertrophic hearts to compensate for the significant decrease in OXPHOS [21]. Despite enhanced glycolysis, various studies showed either decreased or normal glucose oxidation, leading to an uncoupling between the uptake of glucose and its oxidation [19]. If the energy for low work load becomes inefficient, which means deeply impaired contractility, the advanced stage of HF begins which continues to be characterized by repression of FAO and high glycolysis. Here, the use of ketone bodies and branched-chain amino acids (BCAAs) as alternative metabolic substrates are prevailing. These can be further converted to acetyl-CoA to enter the tricarboxylic acid cycle and ETC to produce ATP.

This review aims to describe mitochondrial dynamics and bioenergetics in the heart with references to HF.

## 2. Mitochondria-Related Metabolic Abnormalities in the Failing Heart

**Figure 1:**
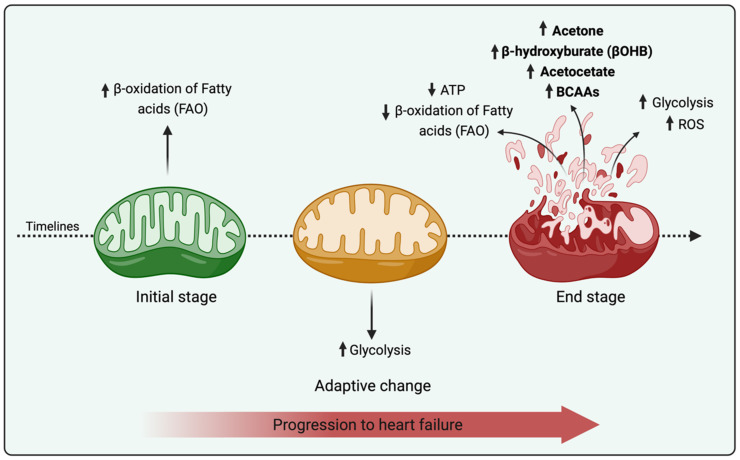
Metabolic and mitochondrial ultrastructural changes in HF. The figure summarizes the first part of the review which reports all metabolic changes and main mitochondrial ultrastructural abnormalities accompanying HF development in humans.

The mammalian heart is considered a metabolic omnivore with the capacity to oxidize fatty acids, ketone bodies, carbohydrates (glucose and lactate), and BCAAs to meet its high energy demand after birth [22]. Cardiac metabolism maintains a dynamic state of equilibrium for efficient energy transfer and is a highly concerted plethora of chemical reactions leading to the conversion of ATP both to sustain cell function and allow contraction, growth, repair, and regeneration. The metabolic alterations in the failing myocardium have been explored; from many points of view [23], HF is considered a return to fetal stages due to the shift from FAO-based metabolism (which mainly manifests as a 35% decrease in the ATP concentration and changes in substrate utilization) to glycolysis (the main active pathway during the fetal period) accompanied by progressive degeneration of the myocardium [24,25] (Figure 1). Accordingly, cardiac metabolism in HF was recognized as a field of active research for well over a century [26] and will be discussed in the first part of this review.

### 2.1. Substrate Changes

#### 2.1.1. Fatty Acids

The beta-oxidation of fatty acids was first demonstrated by Knoop in 1904. FAO represents a predominant fuel source for the myocardium and typically exhibits high flexibility following changes in substrate availability [27]. FAs pass the plasma membrane via tissue-specific transporters and are processed to enter mitochondria for oxidation. Each cycle of FAO produces acetyl-CoA, NADH, and FADH_2_ for ATP production, and this production is estimated to equal 50% to 70% of the ATP consumed during contraction [28]. In HF, a reduction in the expression of genes encoding FAO enzymes was observed in patients and animal models [29], and this decrease is linked to a reduction in mitochondrial respiration; both of these effects are directly correlated with the stage (worsening from early to advanced) and the cause of HF. Accordingly, one of the most consistent metabolic changes in HF is the marked downregulation of fatty acid utilization, which is mainly described in end-stage HF studies [24,30,31] (Figure 1). Cardiac lipid accumulation is also observed in failing hearts from patients who are affected by diabetes and obesity-related metabolic complications, triggering a phenomenon known as “lipotoxicity” [32]. Reduced levels of FAO pathways and the accumulation of incompletely oxidized fatty acids trigger a mismatch between the supply and oxidation of FAs [33]; moreover, the oxidation of glucose and lactates becomes impaired, which leads to an uncoupling of yet increased glycolysis and less cardiac efficiency and function.

In addition to respiratory inhibition, lipotoxicity leads to other mitochondrial dysfunctions caused by a free FAs-dependent drastic permeability of mitochondrial membranes increasing proton conductance [34] and, being long-chain acyl-CoA esters, potent inhibitors of adenine nucleotide translocator (ANT) [35], with serious implications in ROS generation. In the first scenario, free FAs lead to the dissipation of the membrane potential by continuously passing across the IMM from the intermembrane space (IMS) to the matrix and there, releasing protons. Indeed, the matrix has higher pH than IMS. In a second step, FAs come back to the IMS as anions taking advantage of ANT; here, for each of these cycles, the mitochondrial matrix is enriched by one proton. This action would affect membrane permeability and bioenergetics, and trigger cell death [36,37]. Recently, it was demonstrated that introducing omega-3 FA into the diet modifies the composition of free FA, shifting from the precursors of inflammatory states (arachidonic acid) to those involved in their resolution (eicosapentaenoic acid), thus ameliorating cardiac dysfunction [38].

In addition to their impact as energy-providing substrates, FAs act as mediators of signal transduction and as ligands for nuclear receptor peroxisome proliferator-activated receptors (PPARs)-α. The PPAR-α/PXRα complex (retinoid X receptor pathway) and its transcriptional partner Peroxisome proliferator-activated receptor gamma coactivator 1-alpha (PGC1-α) are considered master regulators of mitochondrial biogenesis [39] and FAO by sensing dietary needs and pathological states. Interestingly, in almost all studies in the field, these two compounds were found to be significantly downregulated and thus become the culprits of the metabolic shift toward glycolysis in HF [40,41,42]. In support of this finding, preclinical studies observed repression of OXPHOS, increased oxidative stress, and accelerated HF following pressure overload in cardiac PGC1-α-knockout (KO) transgenic animals.

#### 2.1.2. Glucose

In an effort to counteract the decrease in ATP generation due to OXPHOS impairment, glycolysis rates become elevated in HF [43].

In 1907, Locke and Rosenheim were the first to study myocardial glucose uptake in an isolated rabbit heart model [44]: Glucose and its metabolites play several roles in cardiac myocytes, and their uptake is regulated by the membrane translocation of glucose transporter (GLUT) 1 and GLUT4 followed by phosphorylation into 6-phosphoglucose (G6P). Of note, glycolysis and glucose oxidation are differentially regulated in the heart. Therefore, in failing hearts, the elevated rate of glycolysis is not needed to translate into enhanced glucose oxidation [45]. A plethora of studies support this notion: Patients with HF present enhanced levels of cardiac glycolysis without increases in glucose oxidation, lactate, and pyruvate accumulation [46]. Instead, significant abolition of glucose oxidation was detected in many animal models of HF and in a pool of patients with congestive HF [47] and is currently considered a metabolic marker that proceeds with the development of cardiac deterioration in HF [48]. This feature is due to deregulation of the overall mitochondrial oxidative capacity and alterations in pyruvate dehydrogenase (PHD) activity, which lead to a reduction in the conversion of pyruvate into acetyl-CoA [49,50,51].

Nevertheless, the literature reports apparently conflicting evidence for glucose utilization in HF over time. Additional studies showed that the glucose oxidation rates either remained unchanged in a compensated HF rat model [21] or were elevated in canine cardiac-pacing experiments [41]. The reason for this contradictory evidence is unclear. The most accredited hypothesis is that glucose oxidation varies according to the severity of HF (and to a small extent to the cause); it starts to increase at initial stages and then remains unchanged until it decreases at advanced stages [52].

#### 2.1.3. Ketones

New metabolic changes, including ketone bodies and branched-chain amino acids (BCAAs), were recently identified as alternative substrates in end-stage HF (Figure 1).

Ketone bodies are small molecular energy substrates that are rapidly mobilized and produced during fasting and starvation through hepatic ketogenesis and provide three types of ketones: acetone, β-hydroxyburate (βOHB), and acetoacetate. Ketones can enter cardiomyocytes and be translocated to the mitochondrial matrix, where βOHB is oxidized into acetoacetate by the key enzyme βOHB dehydrogenase (BDH1); thus, acetoacetate is stimulated by succinyl-CoA to acetoacetyl-CoA and then converted to acetyl-CoA by acetyl-CoA acetyltransferase. Consequently, acetyl-CoA is able to enter the tricarboxylic acid cycle and ETC to produce ATP [53,54].

Recent studies by Aubert and associates found that BDH1 was significantly upregulated at least two- to three-fold in an HF mouse model, and this increase is accompanied by significant stimulation of ketone metabolism, which suggests the role of ketone bodies as alternative substrates when glucose oxidation is downregulated in failing hearts [55]. Consistent with previous findings, enhanced myocardial ketone body oxidation rates were found in an ex-vivo-isolated murine heart [56], and significantly higher ketone body levels were detected in the peripheral blood of patients affected by HF than in normal subjects [57]. Although metabolomics studies confirmed that HF utilizes ketones as an alternative substrate in an effort to supply energy at the end stage of human HF, this change does not apport beneficial effects to the evolution of the pathology. Indeed, the plasma ketone concentration and acetone are reportedly linked with malignant prognosis in patients with chronic HF [58,59]. These findings might provide new insights for therapeutic approaches toward HF [60].

#### 2.1.4. BCAAs

Similarly, BCAAs are an important group of essential amino acids and an integral part of myocardial energy metabolism and thus play a crucial role in the pathophysiology of end-stage failing hearts. BCAAs act as key building blocks for peptide synthesis and are effective sources for the biosynthesis of sterol, keto bodies, and glucose. In addition, BCAAs are essential for normal growth and function at the cellular and organism levels [61].

Cardiac BCAA absolute levels were found to be significantly enhanced in a mouse model of HF that underwent transverse aortic constriction [62]. This evidence was correlated with a significant reduction in BCAA catabolic enzymes and in the % of EF, and was associated with insulin resistance [62,63,64]. Moreover, recent studies reported that branched chain alpha-keto acids (BCKAs), which are produced after the initial step of BCAA metabolism, are also increased in the myocardium of HF patients [63]. Enhancing BCAA oxidation in the myocardium can ameliorate the progression of HF by improving cardiac function (increasing the % EF and reverting insulin sensitivity) in a mouse model, which suggests that the imbalance between BCAA availability and use in failing hearts might be correlated with contractile dysfunction [63]. The improved BCCA catabolism was obtained by using 3,6-dichlorobenzothiophene-2-carboxylic acid (BT2), an inhibitor of the branched-chain α-keto acid dehydrogenase kinase (BCKDK) which phosphorylates and inactivates the key enzyme (branched-chain α-keto acid dehydrogenase) responsible for providing substrates used by the TCA cycle [64].

Cardiac BCAA catabolism is described to be deregulated also in myocardial infarction (MI)-operated mice, and this effect contributes to post-MI HF and remodeling by enhancing the mammalian target of rapamycin (mTOR) signaling, a modulator of various anabolic (e.g., protein synthesis) and catabolic (e.g., autophagy) pathways [65]. Alterations in mTOR signaling contribute to the progression of failing hearts [66,67]. Furthermore, the amino acids aspartate and glutamate play a key role in the transfer of reducing equivalents across the mitochondrial membrane for the oxidation of cytosolic NADH through the mitochondrial ETC.

## 3. Mitochondrial Dynamism in the Failing Heart

The development of real-time methods involving the use of chemical dyes and fluorescent proteins or the use of transmission electron microscopy (TEM) has allowed scientists to see mitochondria and study their real morphology, which is highly dynamic and impacts cell function, physiology, and disease [68]. It is interesting to note how several lines of evidence show key roles for mitochondrial fitness in a broad range of cardiac and metabolic disorders, including the development and progression of HF and hypertrophy [69]. In cardiomyocytes, mitochondria are clustered among the myofibrils and the plasma membrane (subsarcolemmal mitochondria or SSM), across the sarcomere myofibrils (intermyofibrillar mitochondria or IMF), and tightly adjacent to the nucleus [70]. Together, these mitochondria are associated with the sarcoplasmic reticulum (SR), a subcellular compartment that participates in cardiac contraction by controlling the processes of Ca^2+^ storage, and release and reuptake in skeletal muscles [71,72].

Overall, the mitochondrial morphology is controlled by known phenomena of balanced fusion and fission that interconnect with each other and to events involving an increased mitochondrial mass (biogenesis) and “in excess” organelle removal (mitophagy) [73]. Whether changes in these pathways are a cause or consequence, or simply accompany the pathology of heart diseases, remains unclear [74].

### 3.1. Ultrastructural Abnormalities

Important ultrastructural abnormalities were detected from an analysis of mitochondria in HF tissues, and these abnormalities, which include the loss of mitochondrial granules, swelling with disorganization of the cristae, vacuolar degeneration, mitochondrial fragmentation, deep structural lesions (disruption of the OMM and IMM and loss of the electrodense matrix), and a strong reduction in the mitochondrial volume [52,75,76,77,78], become increasingly prominent from the initial to end stages of the disease. All of these abnormalities can often contribute to cytochrome c release, which ultimately leads to apoptosis, and were found with both IFM and nuclear mitochondria but not SSM mitochondria [79,80,81,82]. Moreover, the surrounding environment undergoes corresponding changes, including disorganization of sarcomeres and T-tubules.

For correct maintenance of the morphology and structure of the cristae, some proteins located in the IMM that result in being impaired in HF are important; these include mitofilin and cardiolipin [83,84]. Mitofilin is a structural protein, whereas cardiolipin is a phospholipid that is needed to support energy production and regulation of the mitochondrial structure, biogenesis, and dynamics [56,57,58]. Recent studies demonstrated that the downregulation of mitofilin in living cells induces the formation of mitochondria with disorganized inner membranes, loss of mitochondrial function, increased ROS production, and apoptosis [85]. Notably, low levels of mitofilin were also found in tissues of human hearts with HF [86]. However, the downregulation of cardiolipin in HF induces an increase in the production of ROS and mitochondrial dysfunction in cardiomyocytes until death [87]. The maintenance of mitochondrial function through the prevention of mitofilin and cardiolipin is thus important to limit the development of HF.

### 3.2. Biogenesis

As previously mentioned, several studies using animal models of HF showed that the expression of some key regulators of energy metabolism, such as PGC-1α and β, is strongly reduced [88,89,90]. Normally, PGC-1α is a protein with a key role in mitochondrial biogenesis and improves OXPHOS and FAO [91]. Its overexpression, which usually occurs in response to external stimuli (i.e., physical exercise and exposure to cold temperatures), triggers a significant increase in the volume of the whole mitochondrial network. Moreover, PGC-1α is able to bind several transcription factors, including nuclear respiratory factor (NRF) 1 and 2, to activate transcription factor A (TFAM), which is responsible for initiating the transcription of nuclear-encoded proteins with structural and mtDNA replication properties [92]. In response to PGC-1α deregulation, abnormal mitochondrial biogenesis rates were observed in animals with HF and in patients with different etiologies [86]. As opined by Karamanlidis and colleagues, the main mitochondrial dysfunctions (including their reduced turnover) in failing hearts are due to defects in the mtDNA amount and integrity instead of alterations in the gene expression profile [93]. Indeed, in a significant number of failing human hearts, a reduction in mtDNA-encoded proteins derived from a loss of mtDNA was also observed [93,94].

Nevertheless, several kinds of cardiovascular diseases including HF in advanced stages were observed accompanying the course of mitochondrial-based pathologies caused by mtDNA mutations [95,96]. Among those, MELAS (mitochondrial encephalomyopathy, lactic acidosis, and stroke-like episodes) syndrome was observed in concomitance to 3243 A>G mtDNA substitution [97,98].

In addition to its role in mitochondrial biogenesis and its relationship with energy metabolism, PGC-1α can also improve contractility and endothelial function in models of HF [99,100,101,102]. Recent studies performed using PGC-1α-KO mice showed an accelerated development of HF, which was linked to downregulation of OXPHOS genes [90] and deficiencies in the function and reserve of cardiac energy [103,104].

### 3.3. Fusion and Fission Machinery

The process of mitochondrial biogenesis is tightly interconnected with two additional pathways named fusion and fission, and these pathways allow the origin of an interconnected and fragmented mitochondrial network, respectively [105]. It was shown that proteins contributing to mitochondrial dynamics are greatly expressed in the mammalian myocardium, and their ablation is deadly [72,106,107,108,109]. This finding suggests that in cardiomyocytes, mitochondrial dynamism is essential for noncanonical functions governing mitochondrial quality control, Ca^2+^ signaling, cardiac development, and cell death [110].

Although an imbalance in mitochondrial dynamics usually accompanies several cardiovascular diseases, such as ischemia-reperfusion (I/R) and diabetic cardiomyopathy [111], very few studies have addressed these pathways in HF [112]. Mitochondrial dynamics represent continuous events of fission and fusion occurring at all developmental stages of a cell and contribute to cell division, cell death, mtDNA, and nutrient exchange to sustain metabolism in the heart properly [79,80,81,113]. Proteins involved in mitochondrial fusion and fission were highly preserved during evolution [114]; they utilize GTP energy to guide conformational changes and exhibit distinct mitochondrial sublocalization [105]. Mitochondrial fusion is a complex mechanism involving three steps: tethering, OMM fusion, and IMM fusion. Tethering occurs through one or two homologous proteins that belong to the superfamily of mitochondrial transmembrane GTPases. Proteins that are located on the OMM and involved in fusion are mitofusin 1 (Mfn1) and 2 (Mfn2) [115,116]. These mediators can form three different molecular complexes: (i) Mfn1 homotypic oligomers, (ii) Mfn1-Mfn2 heterotypic oligomers, and (iii) Mfn2 homotypic oligomers [117,118]. Fusion of the OMM is normally followed immediately by IMM fusion directed by another dynamin superfamily GTPase, optic atrophy 1 (Opa1), which leads to the final formation of filamentous organelles [119]. This protein is located on the IMM and is redistributed to the IMS. In addition to controlling IMM fusion, Opa1, according to certain protein modifications, can also exert specific effects on the cristae structure [120].

Previous studies showed the essential role of mitofusins during adulthood, which involve conferring protection against long-term cardiac dysfunction, and in early embryonic cardiac development [121]. Unexpectedly, their absence induces mitochondrial fragmentation, and this effect is accompanied by respiratory dysfunction and progressive HF [108]. Additionally, a reduction in Opa1 is representative of mitochondrial variations affected by HF in humans. All these protein changes reflect a population of smaller mitochondria with a truly fragmented network controlled by posttranscriptional events and always associated with apoptosis and consequent loss of functional cardiomyocytes [122,123]. Among all established models of HF, Opa1 is the only protein that always shows a decrease in expression during HF [123]. Indeed, perhaps in an effort to compensate for this loss in OPA1, mitofusins were found to increase following HF.

A report study conducted by Menezes T. and colleagues found that Mfn1 is a substrate target for PKCβII (a Ca^2+^-dependent protein kinase). PKCβII accumulates in the OMM during HF and produces extensive mitochondrial fragmentation following the phosphorylation of Mfn1 at serine 86, which results in a loss of GTPase activity, as detected in both animal and human samples.

Contrary to what is known, Mfn1-dependent fusion in cardiomyocytes fails in response to dysregulation of Ca^2+^ cycling and inefficient cardiac contractility [124]. Being all features that characterize HF, further studies should be performed in in vivo samples to confirm the possibility that targeting Mfn1 expression can be fully considered as therapeutic opportunity.

Mitochondrial fission in mammalian cells is led by the cytoplasmic protein dynamin-related protein-1 (Drp-1), which forms complexes at fission sites on the OMM [125], and fission protein-1 (Fis-1), which encircles the outer mitochondria and promotes the assembly of protein complexes on the OMM [126,127]. Indeed, Fis-1, mitochondrial fission factor (Mff), and mitochondrial dynamics proteins 49 and 51 (MiD49 and MiD51) act as receptors for the recruitment of Drp1 to the mitochondrial surface [128]. Drp1 performs multiple functions, often at the intersection between mitochondrial fission and mitophagy [129]. Drp1 cytoplasm-mitochondria shuttling is crucial for its roles and this is finely regulated by many post-translational modifications including phosphorylation, SUMOylation, palmitoylation, and ubiquitination [129].

In HF models, Fis-1 appears to play a minor role because its levels usually remain unchanged; otherwise, the overall amount of Drp1 tends to be higher in human samples of HF while these data are not confirmed in animal models [123]. In light of this finding, on one hand, Drp1 might also play minor roles in HF, including its crucial direct role in cell death when it is upregulated; on the other hand, post-translational modifications (PTM) rather than changes in the overall amount of the protein may occur in HF. For example, it was observed that Sentrin/SUMO-specific protease 5 (SENP5) is able to deSUMOylate and repress Drp1-dependent mitochondrial fission. SENP5 is upregulated in HF and induces a phenotype of apoptotic cardiomyocytes [130]. However, the most well described PTM for Drp1 is phosphorylation, especially at Serine 616 (S616), which ensures Drp1 translocation at mitochondria and fragmentation. In a mouse model subjected to pressure overload, mitophagy is transiently upregulated in the hypertrophic heart in the first week, in a manner dependent from the phosphorylation of Drp1 at S616. This pathway is not further maintained in later stages of hypertrophy where this repression determines irreversible dysfunctions and HF [131].

### 3.4. Mitophagy

Both under baseline conditions and in response to stress, cells activate a highly regulated mechanism, called mitophagy, to digest senescent and damaged mitochondria [74]. The organelles are engulfed by autophagosomes and are subsequently delivered to lysosomes for degradation. This mechanism is crucial for the maintenance of cellular homeostasis; defects in mitophagy trigger and amplify mitochondrial dysfunction (due to the accumulation of aberrant mitochondria), and this effect is accompanied by the development of cardiomyopathies and ultimately HF with severe contractile dysfunction [132]. This process could be regulated by mitochondrial PTEN-induced kinase 1 (PINK1) and the cytosolic ubiquitin ligase Parkin. Normally, when mitochondria are healthy, PINK1 is imported into the mitochondrial matrix through the translocase of the outer membrane (TOM) complex. In contrast, when a damaged mitochondrion exhibits a loss of mitochondrial membrane potential, PINK1 accumulates on the OMM [133], which results in the recruitment of Parkin from the cytosol to the mitochondrial membrane [134]. However, to initiate Parkin-mediated mitophagy, two events induced by PINK1 are needed. The first event is the phosphorylation of Mfn2, which causes attraction on the mitochondrial surface of Parkin, whereas the second event consists of the phosphorylation of Parkin at Ser65 (ubiquitin-like domain), which increases its E3 ligase activity [135,136]. These steps introduce the ubiquitination of mitochondrial proteins to promote phagosome recruitment and the successive degradation of mitochondrial proteins by the lysosome. Studies suggest that for degradation by the autophagosome, mitochondrial protein ubiquitination via the Lys63 linkage plays a signaling role in the recognition of damaged organelles. In this mechanism, some adaptor proteins, such as NBR1 and p62, bind to ubiquitinated mitochondrial proteins and interact directly with LC3 on the autophagosome, creating a link between mitochondria and autophagosomes [137,138]. In addition, the ubiquitination of mitofusins via Lys48 linkage induces their degradation by the ubiquitin/proteasome system [139] to prevent the fusion of both healthy and damaged mitochondria, which aids the final aim of mitophagy.

From these findings, it is clear that mitophagy is a critical mitochondrial quality control mechanism in myocytes [140,141,142]. Defects in mitophagy associated with the proteins PINK1 and Parkin have negative consequences for cardiomyocytes. Although cardiac function is normal in young Parkin-KO mice, as they age, they accumulate abnormal mitochondria, develop irreversible HF, and show increased cell death following myocardial infarction [141]. Similarly, the absence of PINK1 leads to cardiac mitochondrial dysfunction with a great burst of ROS and irreversible cardiac hypertrophy [140]. The loss of PINK1 in mice also increases susceptibility to pressure overload-mediated HF and I/R injury [140,143]. Similar findings were also documented in humans affected by advanced HF, and these can be characterized by inefficient mitophagy as a consequence of the reduction in the overall PINK1 levels. Although the knowledge of mitophagy as a cause or consequence of HF remains controversial, a recent report suggested that a shift between two isoforms of AMP-activated protein kinase (AMPK) alpha (from 2 to 1) occurs in failing hearts and is responsible for its pathogenesis [144]. Restoring AMPKα2 and thus increasing the phosphorylation of PINK1 at serine 495 increases mitophagy to efficient levels to prevent the progression of HF [144].

Increasing evidence in the last decade highlighted a role also for Parkin- and PINK1-independent pathways in the activation of mitophagy under conditions of stress, as those occurring under I/R [132,145]. These molecular routes would include additional players such as cardiolipin, Bcl2/adenovirus E1B 19 kDa protein-interacting protein 3 (Bnip3), a receptor for LC3-II binding at the OMM and FUNDC1, a protein that under hypoxia becomes phosphorylated at S17 by Unc-51 similar to autophagy activating kinase (Ulk1), and dephosphorylated by phosphoglycerate mutase family member 5 (PGAM5) at S13, triggering mitophagy [145,146]. Moreover, and of great interest, an in vivo model of cardiac ischemia showed the activation of a phosphorylations cascade involving S555 of Ulk1, S179 of Rab9, and S616 of Drp1. These actively participate in the formation of a multiprotein complex in which Ulk1 is phosphorylated by AMPK, indirectly inducing the phosphorylation of Drp1 (and thus mitochondrial fission and mitophagy) via assembling with the Rab9-Rip1-Drp1 axis [147].

The fine balance among all these pathways (biogenesis, fusion, fission, and mitophagy) is crucial either to prevent or overcome injuries following stressful conditions and can thus be considered a promising and feasible therapeutic target [1,25,94]. To understand this fine balance better, increased mitophagy might cause excessive mitochondrial clearance, which would leave the myocytes with too few mitochondria to produce sufficient ATP. During acute cardiac injury, such as MI or I/R, a limited increase in mitophagy could be beneficial to clear damaged mitochondria, but in chronic cardiac diseases, such as HF, sustained upregulation of mitophagy might be harmful [132].

These studies clearly show that the dysregulation of mitophagy has the potential to lead to the accumulation of abnormal mitochondria, contractile dysfunction, and ultimately the progressive loss of myocytes.

### 3.5. Mitochondrial Permeability Transition Pore Complex (PTPC)

Dissipation of the mitochondrial membrane potential, which is the signal for PINK1- and Parkin-dependent mitophagy, is usually caused by permeabilization of the IMM in a phenomenon called mitochondrial permeability transition (MPT). MPT occurs when proteinaceous channels, by a still partially unknown entity, open across the IMM and OMM, which causes uncontrolled fluxes of solutes and thereby osmotic stress, mitochondrial swelling, and pro-apoptotic factor release into the cytosol [148,149,150]. Years of intense research found strong similarities between ATP synthase and PTPC, and, in fact, the latter is currently considered a molecular rearrangement of the dimeric form of ATP synthase into monomers with contributions from a plethora of additional proteins with either structural [151] or modulatory properties [152,153,154], as observed following exposure to stressful conditions [150]. The alteration of the ATP synthase structure (in the putative PTPC conformation) exerts major effects on the disturbance of mitochondrial energy and respiration and hence on cardiac performance. Currently, only little information is available regarding the involvement of the PTPC in HF, and, thus, knowledge on its cause/effect consequentiality remains elusive. Of note, as mentioned above, metabolic changes in HF (especially lipids accumulation) might first contribute to membranes’ permeability and membrane potential dissipation, triggering the opening of the PTPC [155].

The activation of mitochondrial phospholipases in the failing heart reportedly prompts the formation of toxic metabolites in a Ca^2+^-dependent manner. These hydroxyeicosatetraenoic acids induce PTPC opening by Ca^2+^ overload, which further worsens the HF scenario and increases the percentage of nonfunctional mitochondria and cell death [156]. This final endpoint was also suggested by independent groups: The final stages of HF are characterized by increased oxidative stress, increased diastolic Ca^2+^ overload, and episodes of ischemia, all of which prompt abrupt PTPC opening [157]. The pharmacological inhibition of the PTPC, such as by the use of cyclosporin A (CsA), reduced PTPC opening, the dissipation of mitochondrial potential and respiratory deficits, ameliorate the adverse conditions surrounding HF [158].

Another crucial determinant in PTPC opening is ROS. Major sources of ROS in the heart are caused by ETC function, nicotinamide adenine dinucleotide phosphate (NADPH) oxidases (Noxs), and uncoupled NO synthases (NOS). Evidence about the importance in limiting oxidative stress in the heart comes from in vivo studies using transgenic mice with impaired levels of intracellular antioxidants, such as SOD2 [159,160], peroxiredoxins [161], glutathione peroxidase [162], and thioredoxin reductase [163]. In all cases, the alteration of the antioxidant expression correlated with the extent of oxidative stress, with important implications in cardiovascular diseases [164]. In the same way, PTPC opening is also dependent on the action of scavenger enzymes in the cell, especially on the glutathione content and function [165]. This phenomenon was in principle analyzed in single cardiomyocytes and described as a vicious cycle of ROS-induced ROS release (RIRR) [165,166] in which the PTPC opening (triggered by ROS) is essential to generate a ROS burst in contiguous mitochondria, and is associated with a significant dissipation of their membrane potential. The use of selective inhibitors, such as rotenone for complex I of ETC and bongkrekic acid for PTPC, inhibited RIRR, providing the proof of evidence that the ROS burst derives from mitochondria and is generated solely by PTPC opening consequences [165].

Further research on the modulation of PTPC in the failing heart might lead to the discovery of new therapeutic approaches for the treatment of symptoms and regression.

### 3.6. Calcium Cycling and Handling

Ca^2+^ plays an important role in the pathophysiology of the heart [1,167,168]. Its homeostasis is considered an essential mediator in regulating ECC and modulating systolic and diastolic function. Here, Ca^2+^ transduces action potentials in mechanical force. In healthy hearts, the action potential depolarizes the sarcolemma, allowing the passage of low amounts of Ca^2+^ from the extracellular space to the cytosol via L-type calcium channels (LTCCs). As consequence, the magnitude of Ca^2+^ action increases, due to the induced and highly synchronized Ca^2+^ sparks from the RyRs of SR in the whole cell [169,170]. After systole, Ca^2+^ should be removed from the cytosol to guarantee the correct muscle relaxation and, thus, the diastolic phase. SERCA2a, PMCA, and NCX are deputed to do this [171] in an ATP-dependent manner in the first two cases, evidencing further roles and the importance ascribed to ATP in muscle relaxation before a new EC cycle. All these proteinaceous channels are highly regulated by cytosolic proteins which often contribute to the end of the EC coupling but also to pathological states.

SR membranes are closely juxtaposed to mitochondria giving rise to a subcellular compartment named as mitochondria-associated membranes (MAMs) which, in lieu of material exchange, signal transduction and metabolic regulation [172,173]. During ECC it was demonstrated that Ca^2+^ fluctuations occur in mitochondria after SR Ca^2+^ release [174] but they remain controversial if they take part in the regulation of cytosolic Ca^2+^ levels, as it was estimated that mitochondria can handle up to 15% of cytosolic Ca^2+^, compared to other extrusion systems [175]. However, mitochondrial Ca^2+^ uptake has great repercussions on cardiomyocytes fate; in healthy bodies, by ensuring bioenergetics and cardiac contractility [9,10,176], and in disease bodies, where an overload can induce the PTPC opening upon I/R and a vicious cycling of ROS generation, oxidation of RyR2, and SR Ca^2+^ leak in HF, associated with mitochondrial fragmentation and dysfunction [177]. Mitochondrial Ca^2+^ dysregulation was reported also as defects in MAMs’ morphology where the distance among contact sites between SR and mitochondria is increased in stages immediately preceding HF such as hypertrophy, chronic noradrenaline stimulation, and aging [178,179].

**Figure 2:**
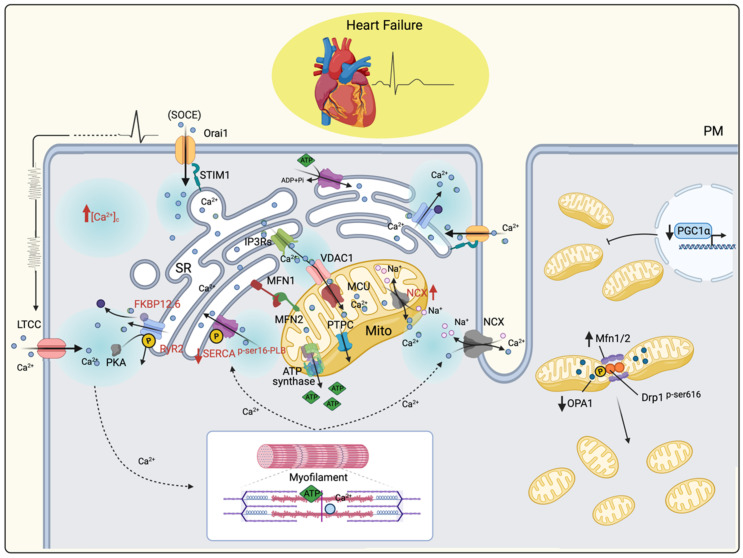
Calcium cycling in the failing heart. The figure summarizes calcium cycling in the normal heart and how it changes in the failing heart. Proteins responsible for these alterations are highlighted in red (e.g., SERCA2a expression and molecular regulation; RyR2 aberrant gating), and the pathways to which they refer are described in the text. The importance of ATP in muscle contraction and relaxion is also depicted. On the right, a graphical abstract highlighting biogenesis and fusion-fission machinery in HF.

Myocytes from failing hearts are characterized by reduced and slower cytosolic Ca^2+^ transients ([Ca^2+^]_i_); indeed, SR Ca^2+^ storage and release are impaired (Figure 2). Although this feature is mainly associated with a reduction in the SERCA2a protein amount, which becomes significantly downregulated in failing hearts compared with nonfailing hearts, its regulation might also play a crucial role because serine 16 phospholamban phosphorylation is reportedly reduced accordingly [16]. Moreover, increased Ca^2+^ leakage from the SR was explored as a secondary cause due to the dissociation of FKBP12.6 from RyR2 following protein kinase A (PKA)-dependent hyperphosphorylation at serine 2808 [180,181] (Figure 2). RyR2 expression does not change in HF but assumes aberrant gating based on the previously mentioned model.

Confirming the undoubtedly essential function of SERCA2a, the restoration of its expression with adeno-associated (AAV) vectors was classified as successful gene therapy in preclinical models of HF [17,182]. Recently, multiple ongoing clinical trials (as reviewed in [183]) are studying the use of AAV to improve SERCA2a expression and function. In addition, pharmacological treatments, such as the use of Istaroxime [184], stimulate SERCA2a and completely restore Ca^2+^ cycling.

The recorded elevation in cytosolic Ca^2+^ load is also caused by an altered influx from the extracellular milieu. Indeed, pathological SR Ca^2+^ depression activates the function of LTCCs and store-operated calcium entry (SOCE), which rewire a notable amount of extracellular Ca^2+^ into the cytosol [185] (Figure 2).

Concomitant with this finding, an additional molecular mechanism was observed in some models of HF, including humans: An increase in NCX function attempts to remove excess cytosolic Ca^2+^ to compensate for inefficient SERCA2a function and also further depresses SR calcium [186,187]. In contrast, other studies found that NCX is impaired by working in a reverse mode due to high [Na^+^] conditions [188]. These combinatorial effects lead to a complete change in the spatiotemporal fluxes of intracellular Ca^2+^, which reflect a markedly defective systolic contraction and diastolic relaxation of the heart [189].

## 4. Epigenetics in HF

In the last decade, and this is still an evolving field of research, evidence showed the involvement of epigenetics signature in HF. Although it is known that many factors affect epigenetics patterns (environmental, diet, lifestyle, pollutants) [190], and, in turn, epigenetics may be the cause of given pathological states (including HF) [191], we would summarize how mitochondrial function intersects with epigenetics in HF. In detail, the consequences of mitochondrial alterations or metabolites produced during the development of HF are emphasized accordingly to the topic of this review.

Epigenetics programs include DNA methylation, histone acetylation/methylation, and noncoding RNAs [192]. Chromatin acetylation involves acetyltransferases and deacetylases which make it more or less accessible to DNA binding elements, respectively. The role of this couple of enzymes was strongly matched to irreversible cardiac remodeling due to either the activation (when acetylated) or depletion (when deacetylated) of GATA Binding Protein 4 (GATA4) and Myocyte Enhancer binding Factor 2 (MEF2), transcription factors responsible for the gene expression rewiring during cardiac hypertrophy [193,194,195]. Chromatin methylations are reversible and can be related to activation and inhibition of the transcriptional activity; Kaneda et al. demonstrated the significant relationship between a triple addition of the methyl group to lysine 4 or 9 of histone H3 and HF [196]. Interestingly, three genes located close to these modifications are RYR2, CACNB2 (encoding for the subunit beta 2 of the voltage-dependent calcium channel), and CACNA2D1 (encoding for the alpha-2 and delta subunits of the voltage-dependent calcium channel complex), responsible for intracellular Ca^2+^ cycling [196]. Instead, DNA methylation is usually synonymous with gene expression “shutdown”. In animal models of pressure overload and cardiac hypertrophy, increased DNA methylation led to significant repression of the transcriptional activity in the left ventricle and allowing for cardiac contractility [197,198]. Otherwise, noncoding RNAs alter gene expression in a wide variety of modes which are not the focus of this review and are reviewed elsewhere [199].

It is clear that changes in metabolism play a crucial role in HF. Additionally, metabolites’ availability influences epigenetics patterns, because most of them act as cofactors for enzymes inducing chromatin modifications and control the transcriptional program as a consequence [200]. Of interest, some of these metabolites are part of those intermediates becoming altered and concurring in HF development; these are ketones (such as βOHB, α-ketoglutarate), Acetyl-CoA, NAD+, FAD+, and mitochondrial ROS.

Acetyl-CoA is one of the main regulators of protein acetylation due to the possibility to supply acetyl groups to enzymes in charge of modifying histones [201]. Thus, the rate of FAO (and its changes during HF) would be associated to either higher or lower gene expression on the basis of the Acetyl-CoA produced [202]. This indeed constitutes a critical factor in cycles of acetylation/deacetylation. βOHB also increases acetylation of proteins being a potent inhibitor of histone deacetylases [203]; this action can lead to beneficial effects in HF due to the activation of neighboring genes such as Forkhead Box O3 (FOXO3), which confers resistance to oxidative stress and sirtuins, already known to have strong cardioprotective roles [204]. Moreover, NAD+, an essential cofactor for many enzymes involved in energy production in cardiomyocytes [205,206] and found to be decreased during HF stages, is also essential as a cofactor for sirtuins function [207]. Sirtuins are a family of proteins able to deacetylate chromatin with crucial roles. In this context, the absence of NAD+ has negative effects on the expression of sirtuins which determine no longer protective roles to counteract HF [208]. NAD+ levels are further finely regulated by Nicotinamide Nucleotide Adenylyltransferase 2 (NMNAT2), whose upregulation protect from hypertrophy [205]. During HF, a significant amount of mitochondrial ROS are produced; as well as the role already addressed, they alter both DNA and histone methylation [200], the first one by oxidizing guanosine in 8-oxo-20-deoxyguanosine and producing as a consequence a hypomethylation state of the DNA; this triggers the onset of inflammation and oxidative stress due to the activation of gene expression involved in those pathways [209]; the second, by reducing S-adenosil metionina (SAM), which is responsible for methylation of histones [210].

## 5. Conclusions and Future Perspectives

HF is caused by a multitude of risk factors and is involved in many cardiovascular pathologies such as coronary artery disease, hypertension, faulty heart valves, and congenital defects. For this reason, it is of great interest and involves a considerable percentage of patients. Cardiology has made great strides worldwide in prevention and patient care, but many steps of basic research are still poorly understood, and translational approaches should be improved. If on the one hand, the metabolic switch and related consequences during the adaptative stages and advanced HF are fairly known, further research about Ca^2+^ cycling, handling, signaling, and interconnections with mitochondrial fusion-fission machinery deserves attention. In particular, a focus on the Ca^2+^ spatio-temporal route and which proteins are involved in it, from the extracellular milieu to the cytosol, would be noteworthy. Then, the contribution of mitochondria as organelles closely related to SR and able to buffer Ca^2+^ to orchestrate a plethora of downstream events would also be noteworthy. Although animal models for the in vivo study of MAMs are lacking, and investigations on patients continue to be limited, efforts in this way may improve the creation of targeted therapeutic strategies to recover mitochondrial and SR function in HF.
